# How does AI-generated assessment compare to expert assessment in simulation-based experiences: Analyzing AI-human interrater reliability

**DOI:** 10.1186/s41077-026-00464-5

**Published:** 2026-10-07

**Authors:** Margaret Brace, Gail Furman, Lisa Diewald, Rebecca Shenkman, Stephanie Felgoise, Tracy Oliver

**Affiliations:** 1 Villanova University M. Louise Fitzpatrick College of Nursing, 800 E Lancaster Avenue, Villanova, PA 19085 USA; 2 Villanova University MacDonald Center for Nutrition Education and Research, 800 E Lancaster Avenue, Villanova, PA USA; 3 School of Professional & Applied Psychology, Philadelphia College of Osteopathic Medicine, 4190 City Ave, Philadelphia, PA USA

**Keywords:** Weight bias, Simulation-based experiences, Artificial intelligence, AI-generated feedback, Tuning AI-generated feedback, Health care professional education, Competency-based education

## Abstract

**Background:**

In the health professions, artificial intelligence simulation-based experiences (AI-SBEs) are increasingly used to evaluate learner performance and provide feedback. Benefits of AI-SBEs include providing instantaneous feedback, allowing more practice opportunities than feasible using traditional methods, and reducing educator burden. However, methods for calibrating AI feedback are underdeveloped.

**Methods:**

This study evaluated the feedback provided by a generative AI-based software program (SimConverse) when assessing simulated patient interactions using the Weight Sensitivity Instrument (WSI), a checklist used to assess learners’ weight bias-related clinical behaviors. A two-phase interrater reliability (IRR) approach was used. First, iterative prompt engineering was guided by AI-human IRR results. Four authors and four volunteers generated 31 interactions in SimConverse. Interaction transcripts were downloaded, and another author with expertise in the WSI manually reviewed and scored the transcripts. Percent agreement and Gwet’s AC1 were calculated to compare AI and expert assessments, and the research team reviewed feedback statements and agreement statistics to identify rubric instructions in need of revision. After revising the rubric in SimConverse, four authors produced 10 additional SBE interactions. Expert and AI assessments were explored. In phase two, the AI-SBE was piloted with 60 medical students in a larger intervention study. A random sample of 30 student transcripts were assessed for AI-human IRR, and students’ comments about the AI-SBE were reviewed.

**Results:**

Of the 11 WSI items, expert and AI agreement was consistently good or very good for 7 items across all rounds (Gwet’s AC1=0.71 and higher). One item initially showed poor agreement (Gwet’s AC1=0.56), but agreement improved after prompt engineering (final Gwet’s AC1=0.89). The improved item assessed whether students made specific statements (asked if weight loss was a priority). Three other items had persistent poor agreement (final Gwet’s AC1=0.42, 0.66, 0.64). These items required broader contextual assessment of the transcripts. Most open-ended student reactions to the AI-SBE were positive.

**Conclusions:**

Findings suggest that AI scoring and feedback within AI-SBEs requires iterative prompt engineering and educator/researcher guidance. Tuning was more successful for content-specific items than context-dependent items. Future work could explore best practices for prompt refinement, particularly for more complex and context-dependent rubric items.

**Supplementary Information:**

The online version contains supplementary material available at https://doi.org/10.1186/s41077-026-00464-5.

## Background

In the health professions, artificial intelligence simulation-based experiences (AI-SBEs) are increasingly used in student education [1]. Students may interact with AI chatbot patients to practice communication skills and then receive AI-generated feedback based on a pre-specified rubric [1]. These AI-SBEs have the benefit of offering learners instantaneous feedback about core competencies and the opportunity to practice skills without increasing educator and standardized patient (SP) burden [2–4]. Educational researchers may also potentially incorporate AI-generated assessments and feedback as data in their studies, reducing time spent reviewing and scoring student performance [5]. AI has limitations, however, including the potential to produce assessments that are incorrect or misleading [6–8]. These errors, and the sense of untrustworthiness they induce, can be detrimental to students’ learning, and to the reliability and validity of AI-generated feedback and scores used for research purposes.

While authors have argued that AI tools should be fine-tuned before using them in educational settings [3], there is not yet a consolidated set of best practices for calibrating and validating feedback and assessment in AI-SBEs in the literature to date. Holderried and colleagues [9] compared AI-human interrater reliability (IRR) for the scoring of 106 student interactions with a patient chatbot, finding good overall agreement (Cohen’s kappa = 0.832) though several items had worse agreement (kappa < 0.6). Ting and Wolffsohn [4] asked students to compare simulation experiences and feedback across two different AI-SBE systems and one in-person human SP SBE, finding that students rated the AI-SBE platform SimConverse as highly as in-person actors but rated a ChatGPT chatbot as worse than SimConverse and actors. Wang and colleagues [8] described the use of prompt engineering (creating more detailed and specific instructions for AI) to enhance the clinical accuracy, adaptability, and the sense of anthropomorphism of ChatGPT-generated standardized patients for medical students’ AI-SBEs simulating history-taking. These examples show a diverse range of methods being explored for AI-SBE calibration and testing, considering prompt engineering, AI-human IRR, or learner experiences. However, there is a gap in the literature detailing methods for calibrating AI feedback, particularly the iterative nature of the process.

Despite growing interest in AI-SBEs, a key unanswered question is whether AI can apply assessment rubrics with the same nuance and consistency as human raters—particularly when items differ in complexity, sensitivity, or required interpersonal judgment. Prior work has reported overall AI–human agreement but has offered limited insight into how agreement varies across specific types of assessment items or how AI scoring can be iteratively calibrated to improve performance. This study addresses that gap by describing a holistic process that our team used, combining dimensions of the methods described above, to prepare and evaluate AI-generated scoring and feedback within an established, commercially available AI-SBE program (SimConverse, Melbourne, Australia). 

## Methods

In this study, we used a two-phase approach to prepare an AI-SBE to offer feedback to students based on an existing rubric. Phase 1 (prompt engineering) and phase 2 (piloting with students) both incorporate calculating AI-human IRR and qualitatively reviewing AI-generated feedback. The rubric and the two phases are described below.

### Instrument: modified WSI

The rubric for evaluating student performance in the AI-SBE was the Weight Sensitivity Instrument© (WSI), a checklist for assessing learners’ weight bias-related clinical behaviors [10]. The 20-item WSI was developed for use in traditional, in-person SBEs in the health professions. The WSI is in checklist format, and instructors can score each item as accomplished by the student or not. The WSI has an established scoring rubric and has shown good content validity (Scale Content Validity Index, S-CVI = 0.91 based on e-Delphi results) and good interrater reliability (IRR) across instructors assessing nursing students (Gwet’s AC1 0.90 to 1.0 for 19 items and 0.50 for one item) [10]. Prior to this project, the 20-item WSI had only been applied to traditional, in-person SBEs [10]. The items of the WSI fall into one of three areas of focus, or domains: communication (e.g., “Asked about current health concerns before talking about weight”), psychological safety (e.g., “Avoided use of labels like obese, morbidly obese, fat to describe the patient or patient’s weight”), and physical assessment (e.g., “Provider chose the right size blood pressure cuff”). Since the AI-SBE would be entirely conversational and not involve a physical exam, all 7 physical assessment items and two other items pertaining to taking a physical weight and nonverbal behaviors were excluded. The modified WSI contained 11 items (see Supplemental Table S1).

### SimConverse

SimConverse is an AI-driven SBE platform that allows educators and researchers to design SBE scenarios and select a rubric for evaluation of learner competency. SimConverse uses proprietary, self-developed Large Language Models (LLMs), and does not use third-party models. Learners engage in simulated conversation with an AI-patient who responds audibly. Following the interview, the learner receives immediate AI-generated feedback based on the instructor-selected rubric. In this study, a simulated patient character was created of a woman living with excess weight seeking a new primary care provider to discuss health concerns, including weight gain. The modified WSI was used as the rubric for scoring AI-SBE encounters. Instructors and researchers accessed transcripts of the AI-SBE interactions, rubric scores, and feedback statements in SimConverse.

### Design

**Fig. 1:**
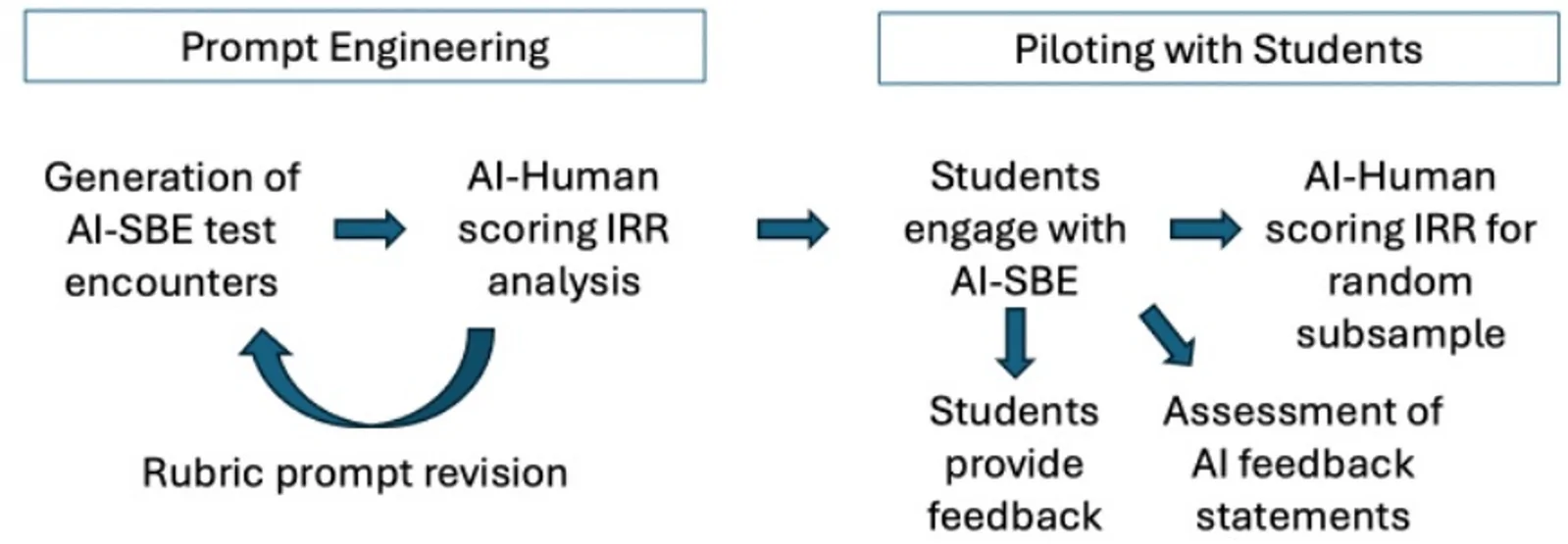
Process for enhancing AI feedback and piloting AI-SBE scoring of weight-sensitive communication

In phase one, the research team engaged in an iterative process of prompt engineering (see Fig. 1). In this project, prompt engineering refers to the revision of the instructions provided to SimConverse for scoring students and providing feedback on the items of the WSI tool. For this, we generated test transcripts for the sole purpose of calibration and prompt engineering. First, four members of the research team and four volunteers generated a total of 31 AI-SBE interactions via SimConverse (1 to 6 interactions per person, median = 4.5). The interactions included gathering information from the patient about her health history and providing her with information on the next steps. The researchers and volunteers producing the interactions ranged from those with no clinical experience to expert clinicians. Transcripts of the interactions were downloaded from SimConverse, and another research team member with expertise in scoring the WSI manually reviewed and scored the transcripts. This reviewer demonstrated good intra-rater reliability (ICC = 0.803) on the WSI, and they were blinded to the AI-generated WSI scores and feedback. AI-human interrater agreement and reliability were calculated (see "Data analysis" section below), and the research team reviewed feedback statements and agreement statistics to identify revisions to the rubric instructions for specific items. For example, the team noted that the simulated patient brought up weight immediately in many encounters and therefore reprogrammed the patient to exhibit more reticence to directly discuss her weight. After revising the modified WSI rubric instructions and patient responses in SimConverse, four research team members produced 10 additional SBE interactions. This second round of interactions included planned attempts to test phrases that had caused disagreement in round 1. Expert and AI assessments were again compared for opportunities to enhance prompt engineering. However, the small sample size maximized efficiency and sacrificed statistical precision, and results should be interpreted as exploratory.

In phase two, the AI-SBE with modified WSI scoring and feedback was piloted with students (see Fig. 1). As part of a larger, one-arm weight sensitivity intervention study, 78 medical students at a single medical school were invited to use the SimConverse AI-SBE before and after receiving weight sensitivity educational content. Specifically, as part of a multi-faceted weight bias reduction intervention embedded in an Interprofessional Education (IPE) curriculum, medical students were asked to view 4 asynchronous e-modules addressing weight bias in health care and best practices for promoting stigma-free weight-related patient conversations. AI-SBEs were incorporated at the beginning and again at the end of the educational series with AI-generated feedback provided to students following each AI-SBE. This study was approved by the authors’ Institutional Review Board, and data were only collected from consenting student participants. A random sample of 30 of the sessions were selected for review by the research team. The same expert reviewer scored the transcripts manually, blinded to AI scores. AI-human IRR was again calculated, and AI-generated feedback for the 30 sessions was reviewed by the research team. Finally, student participants were invited to answer two open-ended questions at the end of the intervention (“What changes or additions to the modules would enhance your learning experience?” and “Any additional feedback you would like to share?”). Comments mentioning the AI-SBE were qualitatively reviewed and coded by two authors.

### Data analysis

The Guidelines for Reporting Reliability and Agreement Studies (GRRAS, see Supplemental Table S2) were followed throughout this manuscript [11]. All quantitative data analysis was completed using StataSE 19.

For AI-human IRR analysis, percent agreement and Gwet’s first order agreement coefficient (AC1) were calculated. Gwet’s AC1 is appropriate for any number of raters and categorical answer choices and has been shown to be more stable than Cohen’s Kappa for checklist-style items that assess presence vs. absence [12, 13]. Like Kappa, Gwet’s AC1 values can be interpreted as follows: <= 0.20 poor agreement, 0.21 to 0.40 fair agreement, 0.41 to 0.60 moderate agreement, 0.61 to 0.80 good agreement, and 0.81 to 1.00 very good agreement. Using the R package *irrCAC* (interrater reliability chance-corrected agreement coefficients), it was calculated that 27 cases would be powered (80%) to estimate an AC1 of 0.80 with a 95% confidence interval of 0.65-0.95 for an item with 2 categories and a prevalence of 0.50. IRR assessments in phase 1 and phase 2 used *n* = 31 and *n* = 30 cases, respectively. For each round, the percent of cases the human rater scored as present (“yes” responses) was calculated, along with the percent of cases the AI rater scored as present. This was done to explore whether one type of rater was scoring more leniently than the other.

Given the small number of open-ended responses, analysis focused on descriptive categorization rather than formal thematic analysis. Two independent coders reviewed all responses and first classified each statement as either positive or a critique of the AI-SBE. Within each category, coders further grouped statements into a small number of descriptive subcategories reflecting the type of comment (e.g., contribution to learning, general positive statements, or specific critiques such as instructional clarity or technical issues). Coders then compared classifications and subcategories and resolved any discrepancies through discussion to reach consensus.

## Results

### Phase 1: prompt engineering

Of the 11 items in the modified WSI, expert and AI agreement was very good for 8 items in the first round, with Gwet’s AC1 values above 0.81 (see Table 1). Three items had moderate AI-human rater agreement (percents agreement 74%, 71%, 68%; and Gwet’s AC1 0.56, 0.50, and 0.50). In each of these cases, AI had a higher percentage of positively scored transcripts than the human rater. The research team reviewed transcripts and AI feedback to develop more detailed instructions to incorporate into the SimConverse rubric. For example, AI initially gave credit to 80.7% of transcripts for any general acknowledgement of behavioral challenges when scoring item 8, (“acknowledged the complexity of managing one’s weight”), rather than recognizing only those statements that addressed the *specific* factors contributing to that complexity. For that same item, the human rater only gave credit to 58.1% of transcripts for accomplishing this task. To improve clarity, examples were added to the WSI rubric prompt indicating that learners should elaborate on the many complex influences on weight beyond diet and exercise and emphasize that managing one’s weight can be very challenging.

**Table 1:** AI-human interrater reliability (IRR) for the 11-item modified Weight Sensitivity Instrument (WSI) across Phase 1 and 2

Modified WSI Items	Phase 1: Prompt Engineering												Phase 2: Pilot					
	**Round 1**,***n*** = 31						**Round 2**,***n*** = 10						**Randomly Selected Student AI-SBEs**,***n*** = 30					
	Human % Yes	AI % Yes	*% Agree*	*AC1*	*(95%*	*CI)*	Human %Yes	AI %Yes	*% Agree*	*AC1*	*(95%*	*CI)*	Human %Yes	AI %Yes	*% Agree*	*AC1*	*(95%*	*CI)*
1. Asked about current health concerns before talking about weight.	74.2	80.7	93.5	0.90	0.75	1.0	80	80	100	1.0	1.0	1.0	93.3	83.3	83.3	0.79	0.58	1.0
2. Asked permission to discuss the patient’s weight.	45.2	38.7	93.5	0.87	0.69	1.0	60	70	90	0.82	0.39	1.0	40.0	43.3	90.0	0.81	0.59	1.0
3. Accepted patient’s preference about discussing weight.	80.7	93.6	87.1	0.83	0.65	1.0	60	90	70	0.52	-0.15	1.0	50.0	66.7	73.3	0.49	0.15	0.82
4. Asked patient’s preference for terms to describe their weight.	19.4	19.4	100	1.0	1.0	1.0	30	30	100	1.0	1.0	1.0	33.3	26.7	90.0	0.83	0.63	1.0
5. Asked about knowledge of the health risks of a high weight.	32.3	25.8	93.5	0.89	0.73	1.0	40	40	100	1.0	1.0	1.0	23.3	23.3	96.7	0.95	0.84	1.0
6. Asked about past experiences with weight or weight management.	58.1	54.8	96.8	0.94	0.81	1.0	50	50	100	1.0	1.0	1.0	36.7	40.0	96.7	0.94	0.81	1.0
7. Asked if weight loss was a priority at this time.	25.8	32.3	74.2	0.56	0.24	0.88	40	50	90	0.80	0.35	1.0	24.1	26.7	90.0	0.84	0.65	1.0
8. Acknowledged the complexity of managing one’s weight.	58.1	80.7	71.0	0.50	0.16	0.83	40	50	90	0.80	0.35	1.0	41.4	46.7	82.8	0.66	0.36	0.95
9. Offered support and partnership.	61.3	93.6	67.7	0.50	0.17	0.84	30	50	80	0.62	0.02	1.0	56.7	76.7	80.0	0.64	0.35	0.93
10. Avoided making negative comments about weight or size.	87.1	100.0	87.1	0.85	0.69	1.0	70	90	80	0.71	0.19	1.0	93.1	100.0	93.1	0.93	0.80	1.0
11. Avoided use of labels like obese, morbidly obese, fat to describe the patient or patient’s weight.	93.6	100.0	93.6	0.93	0.83	1.0	90	90	80	0.76	0.34	1.0	96.6	100.0	96.6	0.96	0.86	1.0

*Human % Yes *Percent of cases that human rater gave credit for accomplishing the item; *AI % Yes *Percent of cases that AI gave credit for accomplishing the item; *% Agree *Percent agreement for 1 human expert and SimConverse AI; *AC1 *Gwet’s AC1 (.81-1.00 very good, .61-.80 good, .41-.60 moderate, .21-.40 fair, <.20 poor); 95%CI is the 95% confidence interval for Gwet’s AC1

After these prompt revisions (and the adjustments to the patient character described in the methods), four authors created another 10 test AI-SBE interactions in SimConverse. Agreement statistics for the three targeted items increased after revision (percent agreement 90%, 90%, 80%; and Gwet’s AC 0.80, 0.80, 0.62, see Table 1). A fourth item (3. “Accepted patient’s preference about discussing weight”) showed a decrease in agreement (round 1 AC1 = 0.83; round 2 AC1 = 0.52). This may have been due to the change in the character to express more reticence about discussing weight. With a sample size of *n* = 10, the confidence intervals for the AC1 values are extremely wide and need to be interpreted with caution. Once again, the AI rater gave credit to more transcripts (90%) than the human rater (60%) for this item. After review by the research team, the SimConverse rubric was revised to clarify that this item should award credit only if the learner sought the patient’s permission to discuss weight and honored the patient’s request. For some examples of prompt revisions, see Supplemental Table S3.

### Phase 2: piloting with students

In phase two, 60 of the 78 invited medical students interacted with the AI-SBE. There were 122 total SimConverse interactions, with two students completing one AI-SBE interaction, 56 completing two AI-SBEs, and two students completing more than two interactions. Demographic characteristics of participating students are summarized in Table 2.

**Table 2:** Characteristics of medical students engaging in at least one AI-SBE encounter in SimConverse

	*N* or M	% or SD
Age (years), M,SD	25.8	2.9
Gender		
Man	13	22.0%
Woman	44	74.6%
Prefer not to answer	2	3.4%
Race and Ethnicity		
Asian	15	25.4%
Black	2	3.4%
Hispanic	5	8.5%
Middle Eastern or North African	2	3.4%
White	30	50.8%
Prefer not to answer	3	5.1%
Multiple	2	3.4%
Class Year		
2nd year	51	86.4%
4th year	8	13.6%
Number of AI-SBE Sessions		
Range	1–5	
Mode	2	

60 students completed any AI-SBE but *n* = 59 for the table due to missing data for one student

Table 1 presents the AI-human IRR results for the random sample of 30 student AI-SBEs. Seven of the modified WSI items had very good AI-human IRR (Gwet’s AC1 0.81 to 1.0), three items had good IRR (Gwet’s AC1 0.61 to 0.80), and one item had moderate IRR (Gwet’s AC1 0.41 to 0.6). Two of the items that were targeted for prompt engineering in Phase 1 had good IRR in this pilot phase (item 8, Gwet’s AC1= 0.66; item 9, Gwet’s AC1= 0.64), and the third improved to very good IRR (item 7, Gwet’s AC1= 0.89) IRR. However, one item remained in the moderate range (item 3, Gwet’s AC1= 0.42). The AI was more likely to give credit for this item than human raters (66.7% vs. 50% respectively), even after the rubric prompts were made more specific and restrictive.

All participating students were invited to offer feedback about their participation in the broader research program, including the AI-SBEs in SimConverse. A total of 28% (*n* = 17) of students who used SimConverse mentioned AI in their comments. Comments were categorized as “positive” or “critiques,” and themes were identified within each group (see Table 3). Most statements were positive (59%, *n* = 10). Several students reported that the AI-SBE contributed to their learning. Other students made general positive comments about AI, and two commented specifically about being impressed with the AI-SBE. The 41% (*n* = 7) of statements offering some critique of the AI-SBE included wanting more instructions for the AI-SBE, wanting a variety of simulated patients rather than a single character, experiencing technical difficulties, or finding the feedback or simulated patient unrealistic.

**Table 3:** Themes from students’ voluntary open-ended comments about the AI simulations (*n* = 17, or 28% of the students who used SimConverse)

Themes	*n*	%	Example
Positive comments	10	59%	
Contribution to learning	4		“I enjoyed the simulations. Even just the action of completing them made me reflect on how and what I should say before I even started the first interview. We have not yet discussed or practiced specific weight concern visits in class yet so I hadn’t really thought about the discussion I would have with patients...”
General positive statements	5		“I loved the AI simulated patient conversations.”
Impressed by AI	2		“...I liked the AI simulation, I was impressed with how well it understood my voice...”
Critiques of AI	7	41%	
Wanting more instructions	2		“A little more guidance about what needs to be covered during the interview would be helpful...”
Wanting more variety	2		“I think that a wider variety of patients would have enhanced my learning experience...”
Technical difficulties	1		“...My microphone was not registering and I was trouble shooting for a long time...”
Critique of feedback	1		“I found some of the feedback from the AI module a little confusing because I didn’t feel like it reflected what I said”
Critique of AI character	1		“The AI generated voices don’t have an expected tone which made the conversation feel eerie...”
Total AI-related comments	17		

The total number of positive comments (10) is less than the count of positive themes (11) because one comment included two themes

## Discussion

When using AI-generated scoring and feedback in AI-SBEs for educational purposes and/or research, iterative testing should be implemented to calibrate AI feedback against human expert feedback. This study found that, for a few items, AI required more nuanced and explicit instructions than originally provided to discern whether learners met the rubric criteria. These refinements were essential because the AI often interpreted broad or superficial statements as sufficient, whereas human raters looked for specific, context appropriate behaviors aligned with weight sensitive communication. Changes to the character’s parameters (increasing reticence to discuss weight) may also have contributed to reduced agreement on at least one item (item 3, “accepted patient’s preference about discussing weight”). Ongoing calibration, therefore, is critical to ensure that AI-generated scoring reflects the depth, specificity, and professional judgment embedded in human developed rubrics.

A notable finding of this study is the clear variation in AI–human agreement across different types of WSI items. Items requiring recognition of specific, concrete behaviors (e.g., asking about weight management history) showed consistently higher agreement, whereas items requiring broader contextual interpretation or sensitivity to patient preferences (e.g., offering partnership or accepting the patient’s preference about discussing weight) demonstrated lower agreement. This pattern highlights that AI does not struggle uniformly; rather, its limitations are concentrated in items that depend on interpersonal cues, understanding the context, or small nuanced details in the conversation. Recognizing this item-level variation is critical for educators and researchers, as it identifies which competencies AI can reliably assess and which require more deliberate prompt engineering or continued reliance on human judgment. This insight reinforces the importance of iterative calibration and provides a practical direction for future refinement of AI-based assessment tools.

In their assessment of students’ interactions with a patient chatbot, Holderried and colleagues speculated that low AI-human IRR observed for certain items stemmed from insufficient specificity in the original prompts [9]. In other words, without more detailed guidance, the AI lacked the precision needed to distinguish between superficial responses and the specific communication behaviors the rubric intended to capture [9]. The inclusion of a pre-testing phase resulted in higher IRR for several items once additional clarifications and examples were incorporated into the prompts. While the majority of the modified WSI items had good or very good inter-rater agreement, issues persisted for a few items. This finding aligns with Wang and colleagues’ conclusion that AI-SBEs need continuing improvement to be trustworthy [8], reinforcing that prompt engineering is an iterative and ongoing process.

The modified WSI items that were the most challenging to successfully prompt were items that required a broader reading of the AI-SBE conversation (e.g., “Offered support or partnership,” “Accepted patient’s preference about discussing weight”). To the contrary, item prompts for identifying whether specific discussion topics were broached were easier to adjust successfully (e.g., “Asked if weight loss was a priority at this time,” “Asked about past experiences with weight management”). These observations support Corbin and colleagues’ [14] argument that AI is particularly fit for providing feedback that is informational with a surface-level personalization (e.g., feedback that does not consider a student’s broader trajectory and context). Future research should investigate prompt engineering strategies for more complex and context-dependent rubric items.

Incorporating AI simulated experiences in real life educational settings requires additional considerations. For scale items demonstrating consistently low agreement between AI and human raters, differences may reflect the challenges inherent in evaluating weight bias rather than shortcomings of the scale itself. Addressing communication skill proficiency often requires attention to context, tone, verbal and nonverbal messaging, and other factors shaping clinical interactions. Some of these nuances may be difficult to capture through AI scoring alone. In actual educational and simulation-based settings, items with poor AI-Human agreement may therefore be better viewed as priority areas for further discussion and reflection rather than as items lending themselves to a clear-cut scoring metric [15]. Used in this manner, AI-generated feedback may be most useful when positioned as one part of a broader, more substantive evaluation process, effectively serving as a starting point for group discussion and learner reflection rather than as a single assessment or proficiency score [16]. This combined approach preserves the importance of human judgment in promoting a deeper understanding of weight bias while complementing it with AI support to support learning objectives [15].

While AI feedback lacks the contextual and relational understanding that a human instructor may bring, students tend to perceive AI feedback as lower risk than instructor feedback [6], which can be beneficial for creating valuable low-stakes opportunities to practice communication skills on sensitive topics and may help extend reach to a wider learner audience. This may be especially important in early phases of training or for low incidence occurrences in clinical practice. Students in this study generally responded positively to their interactions with the AI-SBE platform, with a minority of students mentioning a lack of realism. When authenticity and realism are low, the effectiveness of the AI-SBE can be negatively impacted [1]. However, the realism of the simulated patient can be adjusted with prompt engineering [8], though this was not explored in this study.

This investigation has several limitations. Our sample sizes for IRR analyses were small and allowed for moderately wide confidence intervals, particularly in the second round of phase one. The small samples could contribute to the relative instability of some IRR estimates across rounds, but the small samples were a practical tradeoff to enable the feasibility of the iterative improvement process. The same authors contributed to both generating test interactions and revising the rubric during the prompt engineering phase. While familiarity with the rubric revisions allowed targeted efforts to test the noted weaknesses of the AI assessment, it may have introduced bias or the appearance of bias into the process. The results of this phase should not be interpreted as a test of independent observations. The authors also acknowledge that the differences in competency levels between phase 1 (non-clinicians to expert clinicians) and phase 2 (students at similar levels in their medical training) make direct comparison of IRR metrics difficult. Namely, IRR may look better for Phase 2 due to less variability in participants’ performances. Future work implementing this approach may benefit from using similar participants across phases.

There are several potential limitations to the generalizability of these results. This assessment used the proprietary SimConverse environment, though it is likely that principles of prompt engineering would be similar across other AI platforms as well. Additionally, this study focuses on a specific rubric, the WSI, within an AI-SBE. Weight sensitivity includes more obvious and explicit dimensions, as well as subtle dimensions that can be challenging for even humans, let alone AI, to identify. Finally, our process used AI-SBEs with a single patient character (female who was generally cooperative and friendly) whose character prompt was edited during Phase 1. It is unclear whether other patients and interaction styles (e.g., oppositional) would present different calibration opportunities and challenges. Beyond the specific nuances of weight sensitivity and AI patient characteristics, though, we believe the process described in this paper, which highlights the role of the educator or educational researcher in guiding AI-SBE development and refinement, may be broadly applicable.

Lee and Moore’s systematic review of the use of AI in providing automated feedback in higher education found that many studies fail to discuss the role of the instructors (or educational researchers) in prompt engineering and other aspects of successfully utilizing AI-generated materials in educational settings [7]. AI-SBEs have the potential to enhance the flexibility and personalization of health care education [2], but the success of these AI tools may be a function of educators’ adoption strategies and ethical safeguards for students [17]. Namely, Alghamdi and Alghizzi argue that AI feedback does not replace the judgment of instructors, and instructors need to use their judgment to actively shape AI feedback and integration into education [14].

## Conclusion

Our findings suggest that utilizing AI scoring and feedback within AI-SBEs requires iterative prompt engineering and educator/researcher guidance. Future work could explore best practices for prompt refinement, particularly for more complex and context-dependent rubric items. There is a need for longitudinal research on the impact of feedback in AI-SBE experiences. While preliminary findings are promising, future research should compare student learning outcomes in AI-SBEs with traditional SBEs, as well as translation to clinical practice.

## Supplementary Information

Supplementary Material 1.

## Data Availability

The datasets generated during the current study are not publicly available but are available from the corresponding author on reasonable request.

## Abbreviations

AC1: Agreement coefficient
AI: Artificial intelligence
AI-SBE: Artificial intelligence simulation based experience
CI: Confidence interval
IRR: Interrater reliability
SBE: Simulation based experience
S-CVI: Scale content validity index
SP: Standardized patient
WSI: Weight Sensitivity Instrument©
