# Supplementary material for: How does AI-generated assessment compare to expert assessment in simulation-based experiences: Analyzing AI-human interrater reliability
Source: Adv Simul (Lond). 2026 Oct 7;11:70. doi: 10.1186/s41077-026-00464-5 (PMC13644239; doi:10.1186/s41077-026-00464-5)
Supplement: Supplementary file 1 [file 41077_2026_464_MOESM1_ESM.docx]

**SUPPLEMENTAL MATERIALS**

**How Does AI-Generated Assessment Compare to Faculty Assessment in Simulation-Based Experiences? Analyzing AI-Human Interrater Reliability**

**Table S1.** Original Weight Sensitivity Instrument (WSI) items (Oliver et al., in press), and WSI modified for Artificial Intelligence Simulation Based Experiences (AI-SBE).

|  | **Original WSI** | | **AI-SBE Modified WSI** | |
| --- | --- | --- | --- | --- |
| **Domain** | # | **Item** | # | **Item** |
| Communication | 1 | Provider asked about patient’s current health concerns before talking about weight. | 1 | Asked about current health concerns before talking about weight. |
| Communication | 2 | Provider asked permission to discuss patient’s weight. | 2 | Asked permission to discuss the patient's weight. |
| Psychological Safety | 3 | Provider accepted patient’s preference about discussing weight. | 3 | Accepted patient's preference about discussing weight. |
| Communication | 4 | Provider asked patient’s preference for terms to describe their weight. | 4 | Asked patient's preference for terms to describe their weight. |
| Communication | 5 | Provider asked patient about knowledge of the health risks of a high weight. | 5 | Asked about knowledge of the health risks of a high weight. |
| Communication | 6 | Provider asked patient about past experiences with weight or weight management. | 6 | Asked about past experiences with weight or weight management. |
| Communication | 7 | Provider asked patient if weight loss was a priority at this time. | 7 | Asked if weight loss was a priority at this time. |
| Communication | 8 | Provider acknowledged the complexity of managing one’s weight. | 8 | Acknowledged the complexity of managing one's weight. |
| Communication | 9 | Provider offered support and partnership to patient when discussing the ability to make health behavior changes OR provider made a statement about working as a team. | 9 | Offered support and partnership to patient when discussing the ability to make health behavior changes OR made a statement about working as a team. |
| Communication | 10 | Provider avoided making negative comments about weight/size. | 10 | Avoided making negative comments about weight or size. |
| Physical Assessment | 11 | Provider performed a physical exam. (if no: jump to Q12) | -- | -- |
| Physical Assessment | 11b | Provider performed a physical exam under the gown, on skin, for **each assessment**. | -- | -- |
| Physical Assessment | 12 | Provider measured blood pressure. (if no: jump to Q13) | -- | -- |
| Physical Assessment | 12b | Provider chose the right size BP cuff. | -- | -- |
| Physical Assessment | 13 | Provider measured patient’s weight. (if no: jump to Q14) | -- | -- |
| Physical Assessment | 13b | Provider asked permission to measure patient’s weight. | -- | -- |
| Physical Assessment | 13c | Provider measured patient’s weight in a private space. | -- | -- |
| Communication | 13d | Provider accepted patient’s preference about being weighed (whether to be weighed or not). | -- | -- |
| Psychological Safety | 14 | Provider **avoided** use of labels like obese, morbidly obese, fat, etc. to describe patient or patient’s weight (terms already selected by the patient do not count). | 11 | Avoided use of labels like obese, morbidly obese, fat to describe the patient or patient's weight. |
| Psychological Safety | 15 | Provider **avoided** use of nonverbal behaviors that were disrespectful (eye rolling, sighing, making noises, pointing, etc.). | -- | -- |

**Table S2.** Reporting guidelines for reliability and agreement studies (14)

| TITLE AND ABSTRACT | 1. Identify in title or abstract that interrater/intrarater reliability or agreement was investigated. | Complete |
| --- | --- | --- |
| INTRODUCTION | 2. Name and describe the diagnostic or measurement device of interest explicitly. | Complete |
|  | 3. Specify the subject population of interest. | Complete |
|  | 4. Specify the rater population of interest (if applicable). | Complete |
|  | 5. Describe what is already known about reliability and agreement and provide a rationale for the study (if applicable). | Complete |
| METHODS | 6. Explain how the sample size was chosen. State the determined number of raters, subjects/objects, and replicate observations. | Complete |
|  | 7. Describe the sampling method. | Complete |
|  | 8. Describe the measurement/rating process (e.g. time interval between repeated measurements, availability of clinical information, blinding). | Complete |
|  | 9. State whether measurements/ratings were conducted independently. | Complete |
|  | 10. Describe the statistical analysis. | Complete |
| RESULTS | 11. State the actual number of raters and subjects/objects which were included and the number of replicate observations which were conducted. | Complete |
|  | 12. Describe the sample characteristics of raters and subjects (e.g. training, experience). | Complete |
|  | 13. Report estimates of reliability and agreement including measures of statistical uncertainty. | Complete |
| DISCUSSION | 14. Discuss the practical relevance of results. | Complete |
| AUXILIARY MATERIAL | 15. Provide detailed results if possible (e.g. online) | Complete |

**Table S3.** Examples of initial and revised prompt instructions for WSI items

| **WSI Item Examples** | **Initial Prompt** | **Revised Prompt** |
| --- | --- | --- |
| Asked about knowledge of the health risks of a high weight | The learner asked the patient's knowledge of the health risks of high weight such as asking what have you heard about risk factors, asking do you know the health problems associated with high weight, asking patient's knowledge of impact of extra weight on health. Did the learner ask the patient's knowledge of the health risks of high weight? | The learner asked the patient's knowledge of the health risks of high weight before telling patient about the risks such as asking what have you heard about risk factors, asking do you know the health problems associated with high weight, asking patient's knowledge of impact of extra weight on health. Did the learner ask the patient's knowledge of the health risks of high weight before telling the patient about the risks? |
| Asked permission to discuss the patient’s weight. | The learner asked the patient's permission to discuss weight such as asking if it is ok to talk about your weight, or is your weight something you want to talk about. Did the learner ask the patient's permission to discuss weight? | The learner asked the patient's permission to discuss weight such as asking if it is ok to talk about your weight, or is your weight something you want to talk about, or how do you feel about talking about their weight. Did the learner ask the patient's permission to discuss weight? |
| Asked patient’s preference for terms to describe their weight. | The learner asked the patient's preference for terms to describe their weight. Did the learner ask the patient's preference for terms to describe their weight? | The learner asked the patient's preference for terms to describe their weight, such as how patient refers to their weight, or what patient calls their weight. Did the learner ask the patient's preference for terms to describe their weight? |
| Asked about past experiences with weight or weight management. | The learner asked about the patient's past experience with weight or weight management such as tell me about what you've done in the past to lose weight or what diets have you tried in the past. Did the learner ask about the patient's past experience with weight or weight loss? | The learner asked about the patient's past experience with weight or weight management such as tell me about what you've done in the past to lose weight or what diets have you tried in the past and can include discussion about previous weight struggles or how successful the attempts to lose weight have been. Did the learner ask about the patient's past experience with weight or weight loss? |
| Asked if weight loss was a priority at this time | The learner asked the patient if weight loss was a priority at this time such as is weight loss your goal or what's most important to you or how motivated are you to lose weight? Did the learner ask the patient if weight loss was a priority at this time? | The learner asked the patient if weight loss was a priority at this time before talking about solutions such as asking is weight loss your goal or what's most important to you or how motivated are you to lose weight, or on a scale of 1 to 10 how important is weight loss to you?. Did the learner ask the patient if weight loss was a priority at this time? |
| Avoided making negative comments about weight or size | The learner avoided making negative comments about weight or size such as using the terms obese or obesity or fat when referring to the patient herself or comments that made the patient feel bad or to blame. Did the learner avoid making negative comments about weight or size? | The learner avoided making negative comments about weight or size of the patient such as using the terms obese or obesity or fat when referring to the patient herself (not the condition) or comments that made the patient feel bad or to blame the patient for their weight. Did the learner avoid making negative comments about weight or size? |
